# Epidemiological survey on prevalence and subtypes distribution of *Blastocystis* sp. in Southern Guizhou, China

**DOI:** 10.17305/bb.2024.11303

**Published:** 2024-12-02

**Authors:** Xiaoyin Fu, Jiayin Lyu, Yunliang Shi, Bingying Cao, Dengyu Liu, Xi Yang, Lin Huang, Qiuguo Liang, Dejun Liao, Shanshan He

**Affiliations:** 1 Department of Parasitology, School of Basic Medical Sciences, Guangxi Medical University, Nanning, China; 2 Key Laboratory of Basic Research on Regional Diseases (Guangxi Medical University), Education Department of Guangxi Zhuang Autonomous Region, Nanning, China; 3 Qiannan Medical College for Nationnalities, Duyun, China; 4 The Key Laboratory of Human Parasitic Diseases in Qiannan Prefecture, Duyun, China

**Keywords:** Blastocystis sp., subtype, genotype, ST15

## Abstract

*Blastocystis* sp. is one of the most common intestinal protozoan parasites of humans worldwide and often has genetic polymorphisms. Due to its high prevalence and the possibility of potential transmission to humans, this study was carried out to investigate the prevalence of *Blastocystis* sp. and characterize its subtypes (genotypes) in southern Guizhou, China. A total of 548 fecal samples were collected from hospital patients for culture-based diagnosis. PCR products were sequenced and phylogenetically analyzed to identify subtypes and analyze their distribution. 43 positive cases of infection with *Blastocystis* sp. were detected, resulting in an overall prevalence of 7.85% (43/548). Seven subtypes were identified: ST3 (55.81%), ST1 (25.58%), ST7 (6.98%), ST5 (4.65%), ST2 (2.33%), ST4 (2.33%), and ST15 (2.33%). ST3 demonstrated the lowest intra-ST diversity, followed by ST1. *Blastocystis* sp. infection in southern Guizhou was caused by seven subtypes (ST1–ST5, ST7 and ST15) of the parasite, in which ST3 was the most common subtype, followed by ST1. The lowest intra-ST diversity of ST3 may be associated with substantial interhuman transmission in Guizhou. ST15 was found for the first time in humans, suggesting that it has the potential to be a zoonotic parasite. These findings have enhanced our understanding of the epidemiology and transmission of *Blastocystis* sp. in Southern Guizhou, China.

## Introduction

*Blastocystis* sp. is an intestinal protozoan associated with various human diseases, including urticaria, irritable bowel syndrome (IBS), and ulcerative colitis [1]. However, its pathogenicity remains controversial and requires further investigation [2, 3]. This protozoan is among the most commonly identified parasites in human parasitological examinations, with a global distribution affecting an estimated 1–2 billion people worldwide [4]. Infection rates vary considerably between countries [5] and even within regions of the same country [6]. In humans, the prevalence of *Blastocystis* sp. ranges from 0.5% to 30% in industrialized countries and from 30% to 76%, reaching up to 100% in some developing regions [7]. The global disease burden of this infection is likely significantly underestimated. *Blastocystis* sp. exhibits abundant genetic polymorphisms. Currently, its subtypes are classified using a sequence-based system that analyzes the small-subunit ribosomal RNA (SSU rRNA) gene [8]. This system has identified at least 44 subtypes (ST1–ST17, ST21, and ST23–ST48) [6, 9–11]. Of these, 16 subtypes—including ST1–ST10, ST12, ST14, ST16, ST23, ST35, and ST41—have been detected in humans [9]. The remaining subtypes have been identified only in non-human animal species and are considered to have limited or negligible zoonotic potential [12]. Among humans, ST1 to ST4 are the most common subtypes, while ST5–ST10, ST12, ST14, ST16, ST23, ST35, and ST41 are relatively uncommon to rare [12–16]. Notably, ST3 is the most prevalent subtype worldwide, with its highest prevalence observed in North America (52.9%), followed by Asia (50.5%), Africa (49.8%), Oceania (41.7%), South America (39.2%), and Europe (32.9%) [17]. In continental Europe and the U.K., ST4 is relatively common, but ST1 ranks as the second most prevalent subtype globally [15]. Interestingly, in some developing countries, including Iran, Libya, Nigeria, Tanzania, and Mexico, ST1 has been reported as the most common subtype [18–22]. In China, the prevalence of *Blastocystis* sp. infections varies significantly across regions. The lowest infection rate was reported in the Qinba Mountain Ecological Area (Henan) at 0.04% (3/6706), while the highest was observed in Bama Yao Autonomous County (Guangxi) at 43.26% (215/497) [23]. According to a 2015 survey on major zoonotic diseases in China, Guizhou Province recorded the highest infection rate, at 5.6%, using saline smear and sulfur staining methods. Located in southern China, Guizhou is economically underdeveloped, with a multiethnic population, a mild and humid climate, and widespread parasitic diseases. These factors may contribute to the relatively high prevalence of *Blastocystis* sp. in this region. Currently, knowledge of the prevalence and subtype distribution of *Blastocystis* sp. in Guizhou Province remains limited. Therefore, this study aims to assess the prevalence and subtype distribution of *Blastocystis* sp. in the southern region of Guizhou and to compare these findings with data from other regions of China. The research seeks to enhance our understanding of the transmission risk factors associated with this parasite and provide valuable insights for its prevention and control.

## Materials and methods

### Sample collection and population description

**Figure 1.**
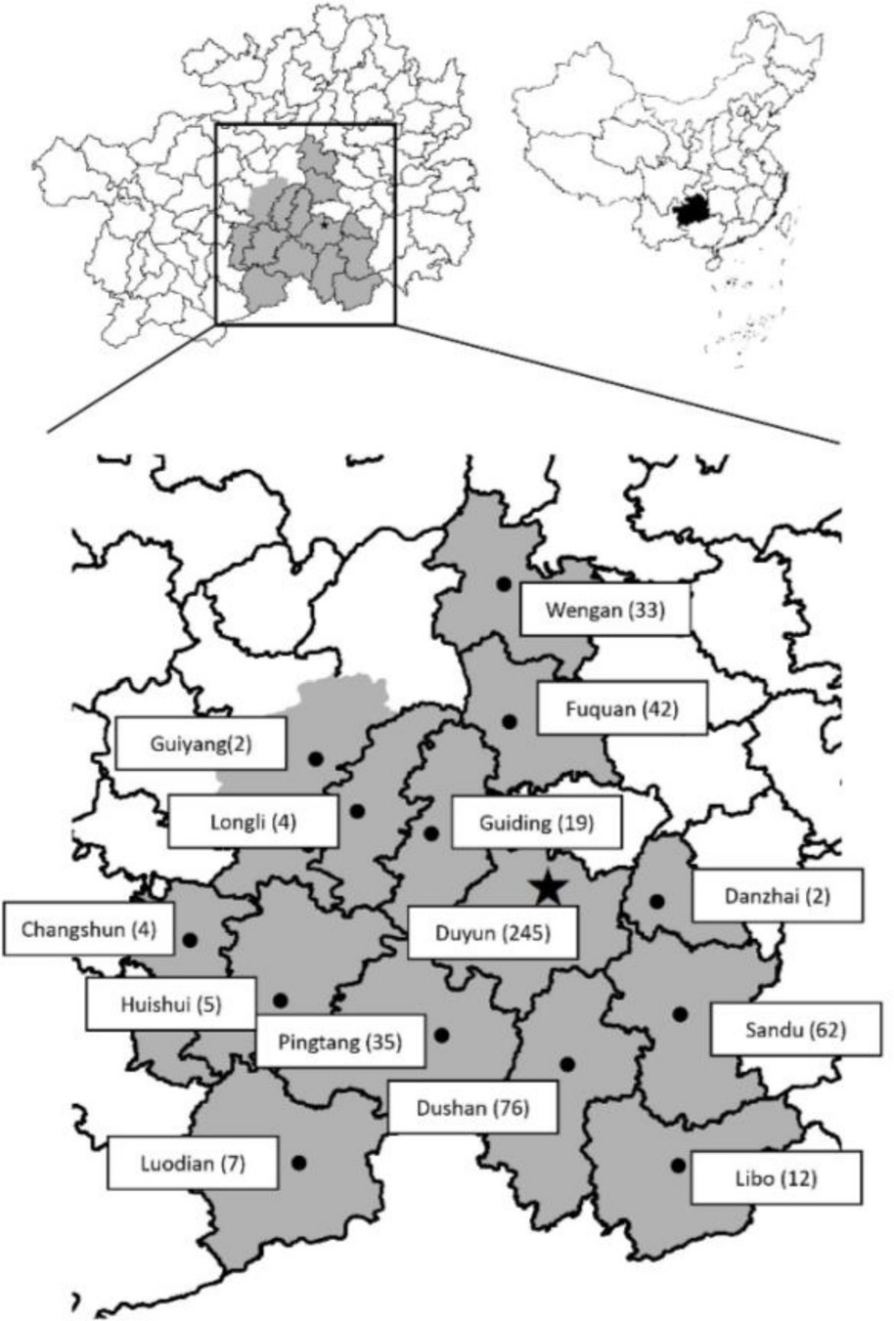
**The map of Guizhou illustrates the residential locations of the subjects participating in the investigation.** The number of participants from each county or municipality is indicated within parentheses. Asterisk represents the locations of hospitals where collected.

The sample collection area, Guizhou Province, is located in the southwestern part of China and features a subtropical humid monsoon climate. Geographically, it spans longitudes 106∘07′ to 109∘35′ E and latitudes 25∘19′ to 27∘31′ N. The province has a total population of approximately 9.62 million, with an urban population of 6.65 million (69.1%) and a rural population of 2.97 million (30.9%). Guizhou is home to more than 40 ethnic minority groups, collectively comprising around 4.36 million individuals (45.3% of the population). Between July and August 2021, we conducted a cross-sectional study at The People’s Hospital of Qiannan in Duyun City, Guizhou Province. Using convenience sampling, we collected stool samples from 548 patients who presented for medical care and required stool examinations. Basic demographic information, including gender, age, ethnicity, occupation, and residential address, as well as clinical data related to gastrointestinal symptoms, urticaria, and IBS, were obtained through the hospital’s Information Management System. Of the participants, the majority (44.7%, 245/548) were residents of Duyun, while the rest originated from 13 additional counties in southern Guizhou. These included Dushan (76 individuals), Sandu (62), Fuquan (42), Pingtang (35), Wengan (33), Guiding (19), Libo (12), Luodian (7), Huishui (5), Changshun (4), Longli (4), Guiyang (2), and Danzhai (2) (Figure 1). Among these 548 patients, the male-to-female ratio was approximately 0.9:1 (259 males and 289 females). The patients were also grouped into four age classes: < 6 years old (*n* ═ 30), 6–17 years old (*n* ═ 22), 18–64 years old (*n* ═ 339) and >65 years old (*n* ═ 157). The median age of attendees was 55 years (range: 3 days–92 years). The patients were from 11 different ethnicities, of which the Han population accounted for 48.18% (264/548), the Miao population accounted for 9.31% (51/548), the Buyi population accounted for 28.83% (158/548), the Shui population accounted for 9.12% (50/548), and the other minorities population accounted for 4.56% (25/548). Among the patients, 41.06% (225/548) were peasants, and 58.94% (323/548) were nonpeasants. Based on their places of residence, 50.55% (277/548) live in rural areas, 13.00% (71/548) lived in townships and 36.45% (200/548) lived in urban area.

### *Blastocystis* cultivation and DNA extraction

All specimens were processed and examined in the parasitology laboratory at Qiannan Medical College for Nationalities. Stool examinations were conducted using culture in Locke-Egg-Serum (LES) medium, prepared through the following methods: Preparation of Normal Locke’s Solution: Dissolve the following components in 1000 mL of distilled water: 8 g of sodium chloride (NaCl), 0.2 g of calcium chloride (CaCl_2_), 0.2 g of potassium hydroxide (KOH), 0.021 g of magnesium chloride hexahydrate (MgCl_2_∙6H_2_O), 5.04 g of disodium hydrogen phosphate dodecahydrate (Na_2_HPO_4_∙12H_2_O), 0.4 g of sodium bicarbonate (NaHCO_3_), and 0.3 g of potassium dihydrogen phosphate (KH_2_PO_4_). Sterilize the solution at 121 ^∘^C for 15 min at 15 psi. After cooling to room temperature, filter the solution through Whatman No. 1 filter paper to remove any precipitates, and then subject it to a second round of high-pressure sterilization. Preparation of Egg Complete Medium: Surface-sterilize fresh chicken eggs using 70% ethanol, then break them into a graduated cylinder. For every 45 mL of egg solution, add 12.5 mL of Locke’s solution. Homogenize the mixture using an ultrasonic bath at 900 W for 3-s intervals (3 s on, 3 s off) for a total duration of 12 min. Transfer 3 mL of the emulsified egg solution into a culture tube, positioning the tube at an angle of approximately 20 mm. Incubate the mixture at 70 ^∘^C for 1 h to allow solidification. After cooling to room temperature, sterilize the tube at 121 ^∘^C for 15 min under 15 psi. Store the prepared medium at 4 ^∘^C for future use. Preparation of LES Medium: To the prepared Locke’s solution, add 10% horse serum, 1000 U/mL of penicillin, 1000 µg/mL of streptomycin sulfate, and 2.5 µg/mL of amphotericin B. Ensure the Locke’s solution fully covers the surface of the egg complete medium (minimum 5 mL) to complete the preparation of the LES medium. Approximately 1 g of each fecal sample was inoculated into the prepared LES medium and cultivated in an anaerobic environment at 37 ^∘^C for 48 h. Iodine staining was used to microscopically observe the vacuolar forms of *Blastocystis*. Infection with Blastocystis was confirmed by identifying vacuolar forms under the microscope in the cultures. Positive *Blastocystis* specimens were centrifuged at 2000×*g* for 5 min in sterile PBS (pH 7.4) to wash the parasites twice. The parasites were then resuspended in 500 µL of PBS for further use.

### PCR amplification

*Blastocystis* sp. was identified via PCR amplification of an approximately 600 bp region of the SSU-rDNA using the barcoding forward primer BhRDr (GAGCTTTTTAACTGCAACAACG) and the barcoding reverse primer RD5 (ATCTGGTTGATCCTGCCAGT) [24]. Each amplification was carried out in a 25-µL PCR mixture containing 12.5 µL of 2× Ex Taq™ Version 2.0 plus dye (Takara, Japan), 9.5 µL of deionized water, 1 µL of each primer (10 µM), and 1 µL of genomic DNA. A positive control (human-derived *Blastocystis* sp. ST3 DNA) and a negative control (distilled water without any DNA) were included in each PCR assay. The PCR conditions were as follows: initial denaturation at 95 ^∘^C for 5 min; 35 cycles of denaturation at 94 ^∘^C for 40 s, annealing at 55 ^∘^C for 40 s, and extension at 72 ^∘^C for 1 min; followed by a final extension at 72 ^∘^C for 10 min. Two microliters of the PCR products were analyzed via 1.5% agarose gel electrophoresis, and the bands were identified based on the expected sizes.

### Sequencing and phylogenetic analysis

PCR amplification products were sequenced by Shanghai Sangon Biological Engineering Technology Service Co., Ltd. (Shanghai, China). The nucleotide sequences obtained in this study were analyzed using BLAST (http://www.ncbi.nlm.nih.gov/blast/), and reference sequences were downloaded from the GenBank database. *Blastocystis* sp. subtypes were identified through BLAST searches (http://blast.ncbi.nlm.nih.gov/Blast.cgi). A phylogenetic tree was constructed using the neighbor-joining (NJ) method in MEGA11 software, with evolutionary distances calculated via the Kimura two-parameter model. Bootstrap analysis with 1000 replicates was conducted to evaluate the robustness of the results. The SSU rRNA sequences from the isolates were compared to all homologous sequences of the known Stentor genus ST using the BLAST on the National Center for Biotechnology Information (NCBI) platform. MEGA11 was subsequently used to assess the diversity within STs and to identify the genotypes.

### Ethical statement

All subjects gave their informed consent for inclusion before they participated in the study. The study was conducted in accordance with the Declaration of Helsinki, and the protocol was approved by the Ethics Committee of Qiannan Medical College for Nationalities (No. 2024024).

### Statistical analysis

All statistical analyses were conducted using SPSS 17.0 software (IBM, Chicago, IL, USA). Differences in the prevalence of *Blastocystis* sp. among individuals of varying sexes, occupations, and home addresses were analyzed using the chi-squared test (χ^2^). Fisher’s exact test was applied to assess associations between *Blastocystis* sp. positivity and factors such as age and ethnicity. A two-sided *P* value of ≤0.05 was considered statistically significant.

## Results

### Epidemiology and clinical characteristics

Out of 548 samples, 43 (7.85%) tested positive for *Blastocystis* sp. using LES medium culture. The prevalence was 9.27% (24/259) in male patients and 6.57% (19/289) in female patients. Age-specific prevalence rates were as follows: 18.18% (4/22) in children under 6 years old, 10.00% (3/30) in individuals aged 6–18 years, 7.64% (24/339) in those over 65 years, and 7.08% (12/157) in the 19–64 age group. By ethnic group, the prevalence was 5.68% (15/264) in the Han ethnic group, 9.80% (5/51) in the Hmong ethnic group, 9.49% (15/158) in the Buyei ethnic group, 12.00% (6/50) in the Shui ethnic group, and 8.00% (2/25) in other ethnic groups. Based on occupation, 7.56% (17/225) of peasants and 8.05% (26/323) of non-peasants tested positive. Regarding regions of residence, prevalence rates were 7.00% (14/200) in urban areas, 5.63% (4/71) in towns, and 9.03% (25/277) in rural areas. However, no significant correlations with sex (χ^2^ ═ 1.369, *P* ═ 0.242), age (Fisher’s exact test, χ^2^ ═ 3.850, *P* ═ 0.241), ethnicity (Fisher’s exact test, χ^2^ ═ 4.364, *P* ═ 0.338), occupation (χ^2^ ═ 1.211, *P* ═ 0.546) or home address (χ^2^ ═ 0.045, *P* ═ 0.832) were found (Table 1).

**Table 1:** Characteristics of the population and of *Blastocystis* sp. positive patients stratified by gender, age classes, ethnic groups, occupations, home addresses and gastrointestinal symptoms

**Variable**	**Positive [*n* (%)]**	**Negative [*n* (%)]**	**χ^2^**	***P* value**	
Gender	Man (*n* ═ 259)	24 (9.27)	235 (90.73)	1.369	0.242
	Female (*n* ═ 289)	19 (6.57)	270 (93.43)		
Age	0∼ (*n* ═ 30)	3 (10.00)	27 (90.00)	3.850^*^	0.241^*^
	6∼ (*n* ═ 22)	4 (18.18)	18 (81.82)		
	19∼ (*n* ═ 339)	24 (7.08)	315 (92.92)		
	65∼ (*n* ═ 157)	12 (7.64)	145 (92.36)		
Ethnicity	Han (*n* ═ 264)	15 (5.68)	249 (94.32)	4.364^*^	0.338^*^
	Hmong (*n* ═ 51)	5 (9.80)	46 (90.20)		
	Bouyei (*n* ═ 158)	15 (9.49)	143 (90.51)		
	Shui (*n* ═ 50)	6 (12.00)	44 (88.00)		
	Others (*n* ═ 25)	2 (8.00)	23 (92.00)		
Occupation	Peasants (*n* ═ 225)	17 (7.56)	208 (92.44)	1.211	0.546
	Non-peasants (*n* ═ 323)	26 (8.05)	297 (91.95)		
Home address	Urban (*n* ═ 200)	14 (7.00)	186 (93.00)	0.045	0.532
	Town (*n* ═ 71)	4 (5.63)	67 (94.37)		
	Rural (*n* ═ 277)	25 (9.03)	252 (90.97)		
Gastrointestinal symptoms	Infected (*n* ═ 43)	3 (6.98)	40 (93.02)	1.911^*^	0.158^*^
	Non-infected (*n* ═ 505)	11 (2.18)	494 (97.82)		

^*^ Fisher’s exact test.

We focused on gastrointestinal symptoms, including nausea, vomiting, abdominal pain, bloating, and diarrhea, as well as urticaria and IBS. The results showed that there was no statistically significant difference in gastrointestinal symptoms between infected individuals (6.98%, 3/43) and non-infected individuals (2.18%, 11/505) (χ^2^ ═ 1.911, *P* ═ 0.158). None of the 548 patients in this study had urticaria or IBS.

### Subtype distribution and phylogenetic analysis

**Figure 2.**
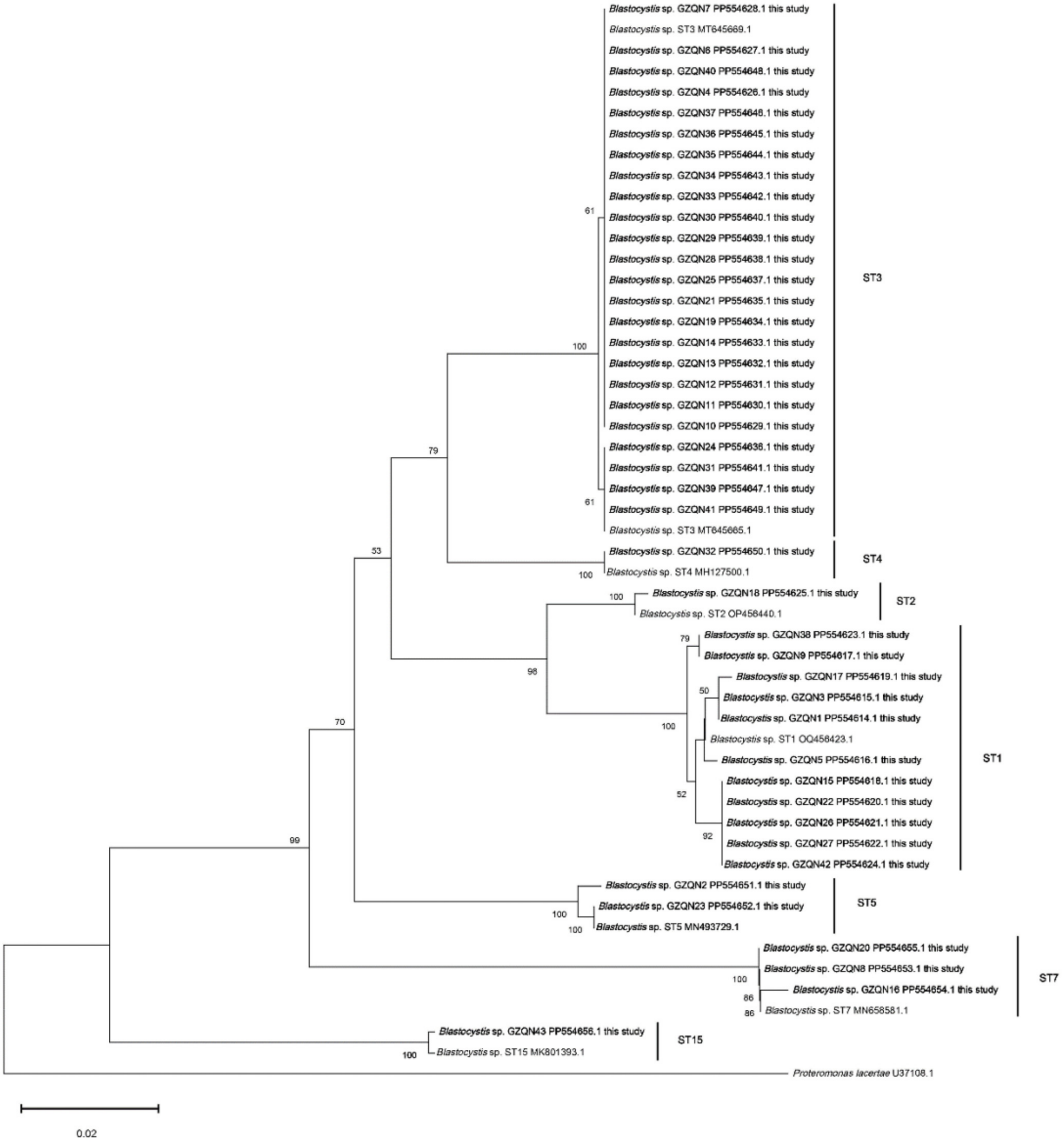
**Phylogenetic relationships among SSU rDNA sequences of *Blastocystis* sp. isolated from the Guizhou population.** SSU rDNA: Small-subunit ribosomal DNA.

**Figure 3.**
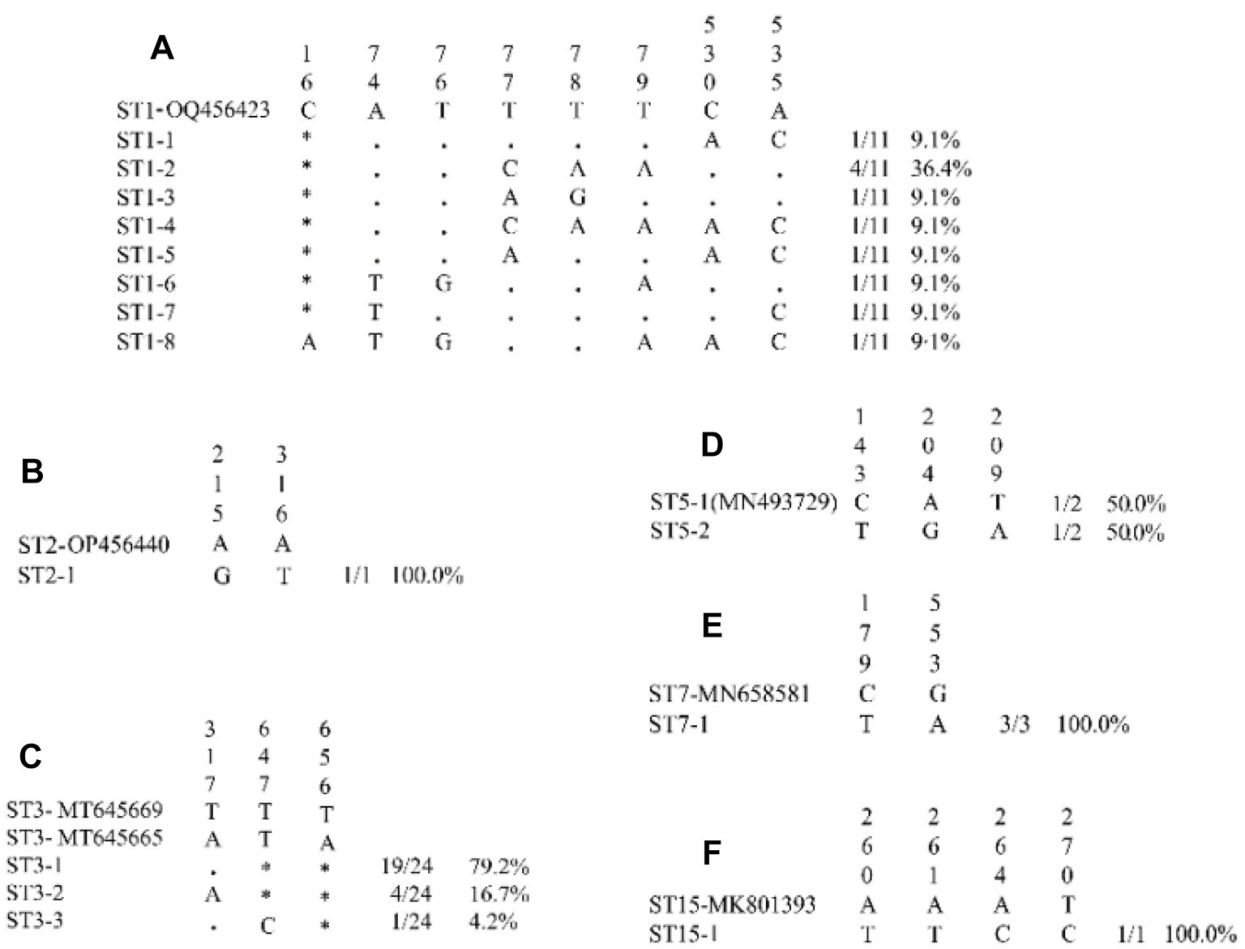
**Alignment of partial SSU rRNA gene sequences from *Blastocystis* sp.** ST1 (A), ST2 (B), ST3 (C), ST5 (D), ST7 (E), and ST15 (F) isolated from the Guizhou population. Variable nucleotide positions were indicated above the alignment (vertical numbering) in comparison to reference sequences [genotypes ST1-OQ456423, ST2-OP456440, ST3-MT645669, ST3-MT645665, ST5-1 (MN493729), ST7-MN658581, and ST15-MK801393]. Nucleotides identical to those of the reference sequences are represented by dots, and gaps are represented by asterisks. Genotypes identified within each ST are shown on the left side of the alignment. The total count and percentage of isolates of each genotype identified in our study are presented on the right side of the alignment. SSU rRNA: Small-subunit ribosomal RNA.

The expected 620 bp fragments of SSU rDNA were successfully amplified and sequenced across all 43 samples. Sequencing identified infections caused by seven subtypes of the parasite: ST1–ST5, ST7, and ST15. Among these, ST3 was the dominant subtype, accounting for 55.81% (24/43) of the identified Blastocystis sp., followed by ST1 (25.58%, 11/43), ST7 (6.98%, 3/43), ST5 (4.65%, 2/43), ST2 (2.33%, 1/43), ST4 (2.33%, 1/43), and ST15 (2.33%, 1/43). Phylogenetic analysis, using the 43 sequences obtained in this study alongside eight reference subtype sequences from GenBank, demonstrated that the sequences clustered with their corresponding reference subtypes (ST1–ST5, ST7, and ST15) (Figure 2). The constructed phylogenetic tree showed that isolates of the same subtype grouped together with strong bootstrap support. To improve our understanding of *Blastocystis* sp. transmission among populations in the central and southern regions of Guizhou, partial SSU rDNA gene sequences from the identified subtypes were compared to evaluate diversity within each subtype. As illustrated in Figure 3, analysis of variable positions within each subtype alignment revealed three ST3 variants (ST3-1 to ST3-3), eight ST1 variants (ST1-1 to ST1-8), and two ST5 variants (ST5-1 to ST5-2). In contrast, only one variant was detected for ST2, ST7, and ST15 (ST2-1, ST7-1, and ST15-1, respectively). Notably, the entire variable region sequence of ST4 was identical to the reference gene, showing no detectable differences.

## Discussion

Previous studies have confirmed that *Blastocystis* sp. infection rates are influenced by several factors, including demographic variations, geographical differences, host immunity, and diagnostic techniques [25–28]. These rates are generally higher in developing countries than in developed ones [29, 30] and more prevalent in warmer, wetter climates compared to colder, drier regions [31]. China, a vast country spanning 9.6 million square kilometers and home to 1.4 billion people, reports the presence of *Blastocystis* sp. across all regions. However, infection rates vary significantly due to differences in topography, climate, economic development, and sanitation across provinces [23]. NING et al. observed that *Blastocystis* sp. infections are more common in rural than urban areas, and protozoan infections are more frequent in southern than northern China [32]. Epidemiological surveys reveal a wide range of prevalence among different populations, from 0.007% to 48.6% [23]. In the current study, the prevalence of *Blastocystis* sp. infection among hospital patients in southern Guizhou was 7.85%, higher than in Shanghai (3.70%) [32] and Heilongjiang (2.4%) [1] but lower than in Guangxi (16.27%–36.6%) [32], Yunnan (9.47%) [32], and Chongqing (10.61%) [1]. Statistical analyses using chi-square and Fisher’s exact tests revealed that the prevalence varies by age, gender, occupation, and ethnicity. *Blastocystis* sp., a protozoan found in the gut, spreads via the fecal-oral route, often through ingestion of contaminated food or water [33]. However, associations between infection and demographic factors, such as age, gender, occupation, and ethnicity remain debated [34]. While some studies show no significant correlations [35–37], others suggest potential associations [38–40]. Overall, there is growing consensus linking infection risk to parasitic exposure. Common risk factors for *Blastocystis* sp. infection include pet ownership, poor hygiene, and consumption of non-sterile drinking water [41–45]. The pathogenicity of *Blastocystis* sp. in humans is controversial [46–48]. Some studies indicate no association between infection and gastrointestinal symptoms [49, 50], while others suggest it as a potential risk factor [51, 52]. Additionally, certain studies link *Blastocystis* sp. to urticaria [53, 54] and IBS [55, 56]. However, our findings revealed no relationship between *Blastocystis* sp. infection and gastrointestinal symptoms. Moreover, since none of the 548 participants reported urticaria or IBS, we could not assess potential correlations between *Blastocystis* sp. infection and these conditions.

Eleven provinces in China—Yunnan, Guangxi, Chongqing, Xinjiang, Jiangxi, Zhejiang, Shanghai, Hebei, Henan, Hainan, and Heilongjiang—have reported cases of *Blastocystis* sp. infections in humans, with geographic variations in the distribution of different subtypes [1, 57–59]. To date, 16 subtypes of *Blastocystis* sp. (ST1–ST10, ST12–ST14, ST17, and ST21) have been identified in China, although only eight of these (ST1–ST7 and ST12) have been detected in the Chinese population [57]. Among these, ST3 and ST1 are the most common, while ST2, ST4, ST5, ST6, ST7, and ST12 are rarely observed [23, 32, 57]. This study identified seven subtypes (ST1–ST5, ST7, and ST15) in the southern region of Guizhou Province. Notably, ST6 and ST12, which were previously reported in China, were not detected. Unexpectedly, we identified a strain classified as ST15 (PP554656) in one patient—marking the first documented case of this subtype in humans, both in China and globally. Previously, ST15 was regarded as a nonhuman subtype, typically found in animals such as domestic pigs (Sus domesticus) and wild boars (Sus scrofa), with ruminants also serving as important hosts [25, 60–62]. Upon reviewing the patient’s case history, we found that they were a local resident of Guizhou Province with no gastrointestinal symptoms at the time of the medical visit, although they had prior contact with chickens, sheep, and other livestock. This case highlights the need for further investigation to determine the potential zoonotic risk of ST15.ST3 and ST1 have consistently been identified as the dominant subtypes in the Chinese population, with ST3 being the most prevalent [23, 57]. In our study, ST3 and ST1 accounted for 55.81% (24/43) and 25.58% (11/43) of cases, respectively, closely aligning with findings from other Chinese provinces. While the distribution of ST3 and ST1 in Guizhou mirrors that of provinces like Heilongjiang, Xinjiang, Zhejiang, Jiangxi, and Shanghai, limited sample sizes and collection areas make it premature to conclude that ST3 is the predominant subtype. Additionally, ongoing debates persist regarding whether ST1 or ST3 is more common in neighboring Guangxi and Yunnan [23, 57, 58, 63–65].

An evaluation of subtype diversity revealed that the 24 ST3 isolates in our study belonged to three genotypes, resulting in an average of 8 isolates per genotype. For ST1, 11 isolates were distributed across 8 genotypes, yielding an average of 1.4 isolates per genotype. The remaining subtypes displayed an average of 1 isolate per genotype. This inverse relationship between intra-subtype variability and the number of isolates per genotype indicates that ST3 exhibits the lowest intra-subtype diversity. Interestingly, ST3 is not commonly found in domestic animals, and its low genetic variability may be associated with ongoing person-to-person transmission within the Guizhou population. In contrast, ST1 exhibits higher genetic diversity, with multiple genotypes identified in animal hosts. This suggests that certain ST1 genotypes may be widely disseminated among populations through environmental contamination involving feces from various hosts.

In our study, we identified genotypes ST2, ST4, ST5, and ST7, which collectively accounted for approximately 16% of the total isolates. Among these, ST2 is reported as the second most prevalent genotype in multiple studies [19, 21, 35, 38, 66]. Molecular epidemiological surveys of *Blastocystis* sp. in individuals from China indicate the presence of ST2 in most provinces, although the proportion is relatively low, except in Hebei Province, where it accounts for 99.74% of cases (389/390) [67]. Consistent with findings from other studies in China, we identified only one ST2 isolate in our study (2.33%, 1/43). The geographic distribution of the ST4 subtype is primarily restricted to Europe [68], with only a few reports from other regions [14]. In our study, we identified a single ST4 isolate (2.33%, 1/43), which aligns with previous findings from studies conducted in China [23, 57]. We also identified two ST5 isolates among the 43 strains. A review of the published literature indicates that ST5 is rarely observed in the Chinese population, with documented cases only in Jiangxi Province (5.77%, 3/52) and Hebei Province (0.26%, 1/390) [57]. Globally, ST5 is also uncommon in humans [69], as it is primarily associated with animal hosts, such as pigs and sheep [70]. However, the potential for zoonotic transmission between humans and animals should not be overlooked. In rural Guizhou, pig farming is predominantly intensive, with centralized management practices. However, pig pens are often located in close proximity to residences. Additionally, in some areas, traditional free-range practices still exist, with farmers allowing pigs to roam in backyards or surrounding forests. These practices can pose significant risk factors for the transmission of ST5. Another relatively rare subtype identified in our study was ST7, with a frequency of 6.98% (3/43). This subtype is primarily found in avian species [32, 69, 70], with only a few reports documenting its prevalence in regional human populations [59]. Guizhou, as one of the most biodiverse regions in China, hosts a wealth of avian resources. It serves as an important migratory route for birds and provides critical habitats for nesting and breeding through its natural reserves and wetlands. With the growth of ecotourism and increasing interest in birdwatching, the potential role of birds in the transmission of *Blastocystis* sp. warrants further consideration.

## Conclusion

*Blastocystis* sp. is a common human intestinal parasite in southern Guizhou. Infection in this region is caused by seven subtypes (ST1–ST5, ST7, and ST15), with ST3 being the most prevalent subtype, followed by ST1. ST3 exhibits the lowest intra-subtype diversity, which may be linked to ongoing interhuman transmission in Guizhou. In contrast, the higher number of ST1 genotypes suggests widespread dissemination among populations through environmental contamination with feces from various hosts. Notably, this study identified ST15 in humans for the first time, indicating its potential as a zoonotic parasite. Further surveys on the prevalence and genotype distribution of *Blastocystis* sp., encompassing individuals from diverse occupations, ethnic groups, and socioeconomic backgrounds, alongside an expanded sample size and representative populations, could provide a stronger scientific foundation for preventing and controlling emerging protozoan diseases in humans.

## Data Availability

The original contributions presented in the study are included in the article material, further inquiries can be directed to the corresponding authors.
